# Role of Shear Stress on Renal Proximal Tubular Cells for Nephrotoxicity Assays

**DOI:** 10.1155/2021/6643324

**Published:** 2021-04-21

**Authors:** Holly H. Birdsall, Timothy G. Hammond

**Affiliations:** ^1^Departments of Otorhinolaryngology, Immunology, and Psychiatry, Baylor College of Medicine, Houston, TX 77030, USA; ^2^Otolaryngology Section, Surgery Service Line, Durham VA Health Care System, 508 Fulton Street, Durham, NC 27705, USA; ^3^Nephrology Section, Department of Medicine, Tulane University School of Medicine, 1430 Tulane Ave, New Orleans 70112, USA; ^4^Space Policy Institute, Elliott School of International Affairs, George Washington University, Washington, DC 20052, USA; ^5^Nephrology Section, Medicine Service Line, Durham VA Health Care System, 508 Fulton Street, Durham, NC 27705, USA; ^6^Nephrology Division, Department of Internal Medicine, Duke University School of Medicine, Durham, NC 27705, USA

## Abstract

Drug-induced nephrotoxicity causes huge morbidity and mortality at massive financial cost. The greatest burden of drug-induced acute kidney injury falls on the proximal tubular cells. To maintain their structure and function, renal proximal tubular cells need the shear stress from tubular fluid flow. Diverse techniques to reintroduce shear stress have been studied in a variety of proximal tubular like cell culture models. These studies often have limited replicates because of the huge cost of equipment and do not report all relevant parameters to allow reproduction and comparison of studies between labs. This review codifies the techniques used to reintroduce shear stress, the cell lines utilized, and the biological outcomes reported. Further, we propose a set of interventions to enhance future cell biology understanding of nephrotoxicity using cell culture models.

The inability to accurately identify nephrotoxicity is a major issue for drug development. Nephrotoxicity is the commonest reason to prolong hospital stays in the United States and elsewhere [1]. Acute kidney injury commonly progresses to end-stage renal disease and the need for renal replacement therapy, e.g., dialysis or transplantation, with its substantial costs and morbidity [2–4]. Fourteen drugs were withdrawn from the market between 1990 and 2010 for nephrotoxicity that had not been detected with available screening strategies [5].

There is currently no FDA-approved in vitro test for nephrotoxicity [6]. A major contributing factor is the lack of a readily available cellular target that is an accurate, representative, and physiologically relevant model of cells in the living kidney. Proximal tubule cells (PTC) are a prime candidate for an in vitro assay of nephrotoxicity. PTC are primarily responsible for the uptake and metabolism of drugs in the kidney [7–9] and are a target for damage from many commonly prescribed clinical drugs including aminoglycoside antibiotics, amphotericin B, radiocontrast media, immunoglobulins, and diverse antineoplastic agents [7, 8].

PTC rapidly dedifferentiates under traditional 2D culture conditions, e.g., 96-well tissue culture plates, which severely limits the utility of this format for in vitro toxicity assays [10]. What PTC need is exposure to fluid shear stress. In vivo, PTC are exposed to fluid shear stress as the blood filtrate from the glomerulus flows past them en route to becoming urine. They sense this shear stress and respond with structural and biochemical changes, changes that need to be maintained in the in vitro environment.

Fukuda et al. found that human primary PTC exposed to fluid shear stress for 24 hours increased their expression of several drug transporters, including SLC37A2, SLS33A2, and SLC47A1 (also known at MATE2-K [11]. Xu et al. found that primary rat tubules maintained their express of P450 CYP1A1 for 12 days if exposed to shear fluid stress in a gyrorotatory culture [12]. Mollet et al. found that HK-2 cells exposed to fluid shear stress in a bioreactor, compared to static cultures, maintained their expression of multiple membrane transporter proteins for 21 days, including PEPT1 (SLC15A1), PEPT 2 (SLC15A2), OCT1 (SLC22A1), OAT3 (SLC22A8), gamma glutamyl transferase (gGT), and sodium-glucose cotransporter-2 (SGLT2) [13]. Unfortunately, none of these authors reported the intensity of the fluid shear stress that was applied to the PTC.

The magnitude of the shear stress to which PTC are exposed is dependent on the quantity of filtrate flowing past, the viscosity of the filtrate, and the internal structure of the proximal tubule. The proximal tubule narrows as one moves distally, the length and density of their microvilli changes, and the composition of the fluid changes as the PTC reabsorb water, proteins, and other components. However, the flow in the initial portion of the proximal tubule can be estimated from the single-nephron glomerular filtration rate of 30 and 90 nL/min [14]. This would suggest that PTC in vivo are exposed to approximately 0.05–0.17 dynes/cm^2^ of shear stress [15] which is much lower than the 5 to 100 dynes/cm^2^ that endothelial cells encounter in the vascular system [16]. These low levels of shear stress are challenging to reproduce in vitro in a uniform manner, particularly when they must be implemented in high throughput applications.

In vitro studies with PTC have used a wide range of shear stresses applied for varying durations, making it impossible to compare results between laboratories. What is needed is a standard uniform method of applying shear stress in vitro that is simple and easy to implement. Two issues have limited studies on shear stress. First, the equipment is usually high-priced, which creates significant capitol barriers to experimentation [17–19]. Second, few techniques to reintroduce shear have thoroughly defined the parameters for reproduction by other labs [20].

This review seeks to categorize the known literature on reintroduction of shear stress on renal proximal tubule cell and the utility of suspension culture models which reintroduce shear to model renal damage. The current aim is to understand the amount of shear induced by different cell culture methods, the cell types utilized, and the outcomes assayed. These insights allow us to recommend interventions in the field of drug-induced nephrotoxicity to move the field forward. We performed PubMed searches using the terms renal proximal tubular cells, suspension culture, bioreactor, proximal tubule, renal cell shear stress, nephrotoxicity, drug toxicity, acute tubular necrosis, and renal genomics. We identified 25 papers that used PTC (or PTC cell lines) and specified the intensity of the shear stress flow and the duration of the stimulus.

Figure 1 compares the intensity and duration of shear stress applied to PTC in these 25 publications. The marker shape indicates the method utilized to generate the shear stress, most of which are microfluidics and parallel plate studies. Each study is referenced with a number, defined in Table 1. This graph gives a stark account of why the field has not come to a universal model for studies of nephrotoxicity: the miscellany of cell types, shear levels applied, and duration of exposure defies simple interpretation.

Fluid shear stress induces structural changes in PTC (Table 1). Reorganization of actin fibers and the cytoskeleton was frequently observed, particularly in experiments using higher intensities of fluid shear stress. The studies that applied higher intensities of shear stress are useful as elevated levels of shear stress on PTC have been implicated in the progression of renal disease [41–45]. Increased expression of microvilli in the presence of fluid shear stress was also noted by multiple authors (Table 1). This is critical, as the microvilli are the sensors for tubular flow and shear stress [23, 29, 31, 46, 47].

Of critical importance to the development of an in vitro nephrotoxicity assay, fluid shear stress also increases the quantity and/or activity of transporters that take up proteins and drugs (Table 1). Exposure to fluid shear stress causes PTC to express more megalin and cubilin, transporters that are central part of the proximal tubular uptake of albumin, many other proteins, and drugs [48–50]. Indeed, albumin transport increases when PTC are exposed to fluid shear stress (Table 1). Renal cells employ a variety of organic anion transporters (OATs) and organic cation transporters (OCTs) [51] in the uptake and secretion of drugs. Fluid shear stress has also been observed to upregulate many of these on PTC including MATE (SLC47A1), OCT2 (SLC22A2), P-gp (ABCB1 or MDR1), MAT2K (SLC472K), and MRP2/4 (ABCC2/4). Jang et al. noted that the P-gp efflux by human primary PTC exposed to 0.2 dynes/cm^2^ shear stress in vitro was closer to that observed in vivo compared to PTC in static 2D cultures [27].

With a specific focus on suspension culture and shear stress effects on renal proximal tubular cells, this review expands and enhances a specific segment of the Good Cell Culture Practice (GCCP) initiative [52] started by the former European Center for the Validation of Alternative Methods (ECVAM) [53]. The GCCP program tries to define standardized protocols to cultivate all relevant human tissues/organs to test toxicity of newly developed drugs and chemicals.

The diversity of shear stress levels and durations in the studies reviewed here emphasizes the need for systematic reporting of specific criteria in order to produce a knowledge base to support harmonized protocols. Our lab proposed this more than a decade ago, embodied in the Bonn criteria [20]. While there are developments on the way to generate harmonized protocols that should allow for prediction of nephrotoxicity during the preclinical phase of drug development [54–56], each methodology will need the kind of summary review presented here to allow useful progression of the initiatives.

The duration of shear exposure and cell type have striking effects on cellular responses (Table 1). It is a conundrum to compare studies not only because of differing shear stress, duration, and cell types, but the various studies utilized diverse outcome measures. A few studies have examined different shear levels and demonstrated changes dependent on shear levels [21, 29, 31]. There is scant, if any, data on the time course of changes in selected outcomes. Hence, study of changes in outcomes over time is one of our suggestions for future study.

Only with harmonized protocols that define the shear stress applied on renal proximal tubules can many of the questions pivotal to predicting nephrotoxicity be answered: does shear induce certain specific patterns in gene expression? Is there an interdependency between the magnitude of shear stress and the expression of specific genes? How do the effects of cells exposed to shear stress or under microfluidic conditions compare? Can the change in phenotypic function of proximal tubular cells in culture towards an in vivo equivalent state be achieved by shear stress alone?

## 1. Conclusions

We propose three strategies to move the field towards a uniform model to test nephrotoxicity of drugs.

First, in 2010, we proposed a minimum data set to be reported to allow reproduction of suspension culture studies in other labs [20]. As the meeting where the proposal was presented was in Bonn, we termed these the Bonn criteria [20]. They include the vessel diameter, rotation speed, media viscosity, media density, cell/organoid/spheroid diameter, and density. The Bonn criteria remain critical to interpret data between labs and allow accurate experimental reproduction between labs.

Second, if different labs continue to use different techniques and reagents including cell types, some tradeoff or bake-off studies will be indispensable to understand differences between approaches.

Last, the development of a low capital, inexpensive, to-use suspension culture technology would allow far more labs access to the technology and occasion the opportunity for studies to include more replicates and conditions cost-effectively.

The search for a uniform model to study nephrotoxicity is severely limited by the use of a multiplicity of methods and techniques, which cannot be simply compared. Laboratories use different cell types approximating renal proximal tubular cells and apply diverse shear stress methods, and there is no systematic and adequate reporting of culture parameters. The morbidity, mortality, and cost of drug induced acute renal injury should make an integrated cell biology approach to nephrotoxicity an urgent priority.

## Figures and Tables

**Figure 1:**
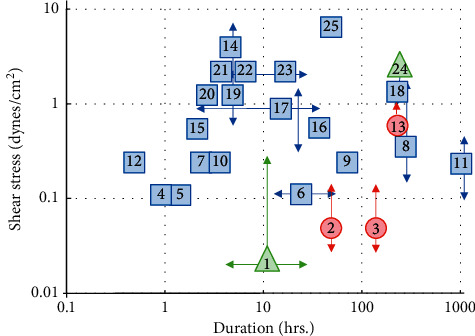
Varying conditions used to expose PTC to shear stress in vitro. The graph illustrates the varying intensity and duration of shear stress applied to cultured PTC from 25 reports in the literature. The *y*-axis is the intensity of shear force in dynes/cm^2^, and the *x*-axis is the duration of the exposure in hours. When a report used multiple of conditions, arrows indicate the range of intensities, and/or times and the marker is placed at the average value. Each publication is indicated with a number which corresponds to the citation in Table 1. The marker shape indicates the method used to apply the fluid shear stress: blue squares are parallel plates and microfluidics, red circles are rotating wall suspension culture, and green triangles are stirring bioreactors and orbital shakers.

**Table 1:** The study reference, shear stress in dynes/cm^2^, duration in hours, technology utilized, and cell type.

Reference number	Shear stress (dynes/cm^2^)	Duration (hours)	Fluid shear stress generated with	Cell	Response of PTC to fluid shear stress			
					Reorganized actin and cytoskeleton	Increased microvilli	Increased cubilin/megalin, albumin transport	Increased expression of drug transporters
1. Bhat 1995 [21]	0.02–0.27	12–15	Spinner flasks with stirrers	MDCK (canine)	**X**			
2. Cowger 2000 [22]	0.04–0.12	48	Rotating wall vessel suspension	Primary PTC (human)				
3. Hammond 1999 & 2000 [20]	0.04–0.12	144	Rotating wall vessel suspension	Primary PTC (human and rat)				
4. Raghavan 2014 [23]	0.1^∗^	0.25–0.5	MIcrofluidics	LLC-PK1 (pig), OK (possum)			**X**	
5. Miravete 2011 [24]	0.5–5	1	Parallel plate	HK-2 (human)				
6. Shimony 2008 [25]	0.1	24–48	Slow rotation)	MDCK (canine), HK-2 (human)				
7. Xu 2020 [12]	0.2	2.5	Microfluidics	HK-2 (human)			**X**	
8. Jayagopal 2019 [26]	0.2–2	240	Parallel plate	MDCK (canine)				MATE & OCT2
9. Jang 2013 [27]	0.2	72	Microfluidics	Primary PTC (human)		**X**	**X**	P-gp
10. Duan 2010 [28]	0.2	3	Parallel plate	Primary PTC (murine)	**X**			
11. Homan 2016 [29]	0.1 – 0.5	1008	Perfused 3-D construct	PTC-hTERT1	**X**	**X**	**X**	
12. Carrisoza-gaytan 2014 [30]	0.2	0.5	Parallel plate	mpkCCD (murine)	**X**			
13. Kaysen 1999 [31]	0.5–1	240–384	Rotating wall vessel suspension	Primary PTC (rat and human)		**X**	**X**	
14. Brakeman 2016 [32]	0.5–5	5	Microfluidics	Primary PTC (human)				
15. Frohlich 2012 [33]	0.5	2	Parallel plate	HK-2 (human)	**X**			
16. Fukuda 2017 [11]	0.5	24–48	Parallel plate	Primary PTC (human)				MAT2K
17. Essig 2001 [15]	0.04–0.17	2–24	Parallel plate	Primary PTC (murine) and LLC-PK1	**X**			
18. Vriend 2020 [34]	0.5–2.0	216	Microfluidics	Immortalized hu PTC			**X**	MRP2/4 and P-gp
19. Duan 2008 [35]	1.0	5	Parallel plate	Primary PTC (murine)	**X**			
20. Ferrell 2012 [36]	1.0	3	MIcrofluidics	Primary PTC (murine)			**X**	
21. Kunnen 2017 [37]	1.9	4–20	Parallel plate and cone-plate	SV40 transformed PTC (murine)	**X**			
22. Cattaneo 2011 [38]	2.0	6	Parallel plate	MDCK (canine)	**X**			
23. Kunnen 2018b [39]	2.0	4–16	Parallel plate	SV40 transformed PTC (murine)	**X**			
24. Ferrell 2018 [40]	2.0	6–240	Orbital shaker	Primary PTC (human)	**X**			
25. Maggiorani 2015 [41]	5.0	48	Parallel plate	HK-2 (human)	**X**			

^∗^Initially reported as 1.0 dynes/cm^2^; corrected in errata to 0.1 dynes/cm^2^. This table displays the effects of fluid shear stress on PTC in vitro. The data are abstracted from 25 publications that reported the amount of shear stress applied (dynes/cm^2^) and the duration of the stimulus. When a range of intensities or exposure times was used, arrows indicate the range and the symbol is placed at the average amount. The PTC cell types used included primary cells (from human, mouse, or rat), MadinDarby Canine Kidney (MDCK) cell line, Human papilloma–transduced PTC (HK–2), SV40–transformed murine PTC, LLC–PK1 (pig kidney line), and OK (opossum kidney cell line). The reference numbers are the key for Figure 1.

## Data Availability

All articles cited are freely available on PubMed and other academic media.

## Abbreviations

gGT: Gamma glutamyl transferase
MATE: Multidrug and toxin extrusion protein
MDCK: MadinDarby Canine Kidney
Pgp: P glycoprotein transporter
PETP: Peptide transporter
PTC: Proximal tubule cells
OAT: Organic anion transporters
OCT: Organic cation transporters.
